# Small Target Recognition and Tracking Based on UAV Platform

**DOI:** 10.3390/s22176579

**Published:** 2022-08-31

**Authors:** Xiangrui Tian, Yinjun Jia, Xin Luo, Jie Yin

**Affiliations:** College of Automation Engineering, Nanjing University of Aeronautics and Astronautics, Nanjing 210016, China

**Keywords:** intelligent recognition, target tracking, deep learning, multi-rotor UAV

## Abstract

Target recognition and tracking based on multi-rotor UAVs have the advantages of low cost and high flexibility. It can monitor low-altitude targets with high intensity. It has great application prospects in national defense, military, and civil fields. The existing algorithms for aerial small target recognition and tracking have the disadvantages of slow speed, low accuracy, poor robustness, and insufficient intelligence. Aiming at the problems of existing algorithms, this paper first makes a lightweight improvement for the YOLOv4 network recognition algorithm suitable for small target recognition and tests it on the VisDrone dataset. The accuracy of the improved algorithm is increased by 1.5% and the speed is increased by 3.3 times. Then, by analyzing the response value, the KCF tracking situation is judged, and the template update of the adaptive learning rate is realized. When the tracking fails, the target is re-searched and tracked based on the recognition results and the similarity judgment. Finally, experiments are carried out on the multi-rotor UAV, and the adaptive zoom tracking strategy is designed to track pedestrians, cars, and UAVs. The results show that the proposed algorithm can achieve stable tracking of long-distance small targets.

## 1. Introduction

The tracking and recognition system of UAVs based on multi-rotor design has the advantages of low cost, good concealment, high flexibility, and small volume. It can make up for the lack of satellite acquisition of near-ground and low-altitude information [1]. Therefore, multi-rotor UAVs have been widely used in civil and military fields. In the military field, multi-rotor UAV recognition and tracking can be used for reconnaissance and tracking of ground military targets, and can also carry weapons to attack targets independently [2]. After finding the target through the recognition and tracking algorithm, the multi-rotor UAV can maintain a safe distance for covert tracking and reconnaissance of the target. Compared with the traditional way of tracking vehicles or personnel, the UAV can achieve low-cost and zero casualty tracking. Because the UAV has a broad field of view from the top of the air, it can better track the target. In the civil field, after fusing the target recognition algorithm, UAVs can be used to monitor the growth of crops [3], rescue and disaster relief, search and rescue trapped people, and also can be used to track and hunt criminals when they escape [4], which has a very good application prospect.

At present, target recognition and tracking tasks are mainly oriented to natural scene images and have been relatively mature in related application fields, such as face recognition, pedestrian detection, and so on. However, due to the different imaging angles of UAVs, the effect of directly applying the existing algorithms to the field of UAVs is poor. Therefore, it is of great significance to study the target detection and tracking algorithm suitable for UAVs [5]. A major feature of traditional target detection algorithms is that the features used are artificially designed features, such as scale-invariant feature transformation (SIFT) [6], histogram of oriented gradients (HOG) [7], and so on.

However, the detection accuracy of traditional methods is poor. In 2012, the team led by Professor Hinton designed AlexNet using convolutional neural network (CNN) and won the championship on image net, making CNN gradually become one of the most important tools in the field of computer vision [8]. Its basic principle is to use CNN to extract deep image features, to achieve target classification and location. At present, target detection algorithms based on deep convolution neural network are mainly divided into two categories. One is to search candidate regions through Selective Search [9], Edge Boxes [10], and other algorithms, and then carry out feature extraction and target detection. Representative algorithms include R-CNN, Fast R-CNN, and Faster R-CNN [11,12,13]. These algorithms have high precision, but slow speed, and are difficult to be improved and applied to UAV target detection in real-time. The other is to directly carry out feature extraction to realize target classification and bounding box regression. Representative algorithms include the YOLO [14] series algorithm and the SSD [15] algorithm. This kind of algorithm has fast speed, but low precision, which is more consistent with UAV target detection with high real-time requirements. The improved algorithms based on YOLOv1 include YOLOv2, YOLOv3, etc. [16,17]. With the proposal of YOLOv4 [18], aiming at the problem of accuracy or real-time performance of the above algorithm, it is of great significance to study the aerial target recognition algorithm of multi-rotor UAVs based on improved YOLOv4.

The research field of target-tracking algorithms started late and develops slowly compared with the vision research direction such as target recognition. Modern tracking methods are based on Deep Learning. By constructing an effective depth model based on a neural network, effective image information is extracted, and a classifier is generated to achieve the effect of target tracking. The relevant algorithms include DCFNet [19], ECO [20], SiamFC [21] and other algorithms. This kind of method has high hardware requirements and slow speed. The traditional methods mainly include the mean shift algorithm, optical flow method, and tracking algorithms based on a correlation filter. In the method based on correlation filtering, the process of target tracking is approximately regarded as the process of correlation filtering on the image of the search area [22]. In the initialization process, according to the samples of the first frame, a discriminative filter is trained based on the minimum mean square error of the output result, to effectively distinguish the background and target. In the process of tracking, the position of the target is found through the response image of the search area after the action of the filter. The larger the value of the response image, the greater the correlation between the image of the position and the target. After the first correlation filtering method, Minimum Output Sum of Squared Error (MOOSE) [23], a large number of methods based on correlation filtering have been proposed one after another. In response to the problem of insufficient MOSSE method samples, Henriques et al. proposed a CSK (Circulant Structure with Kernels) algorithm based on cyclic dense sampling in 2010 [24]; Henriques et al. introduced kernel functions into trackers in 2014 to propose the Kernelized Correlation Filter (KCF) algorithm [25], while extending the original single-channel correlation filter to multi-channels. Correlated filters have higher tracking speeds, while KCF has higher accuracy and speed.

Aiming at the problem of low altitude small target real-time recognition and tracking from the perspective of UAV, this paper proposes a target recognition and tracking algorithm based on the fusion of improved YOLOv4 and KCF. Firstly, the YOLOv4 network is improved to adapt to the small target from the perspective of UAV, then the obtained model is pruned and compressed, and combined with KCF to realize real-time target recognition and tracking. Finally, based on DJI M210 RTK V2 multi-rotor UAV, the algorithm is transplanted to the onboard computer Manifold-2G and combined with DJI-OSDK to realize UAV recognition and tracking of interesting targets.

## 2. Recognition Algorithm Based on YOLOv4

### 2.1. YOLOv4 Algorithm

**Figure 1:**
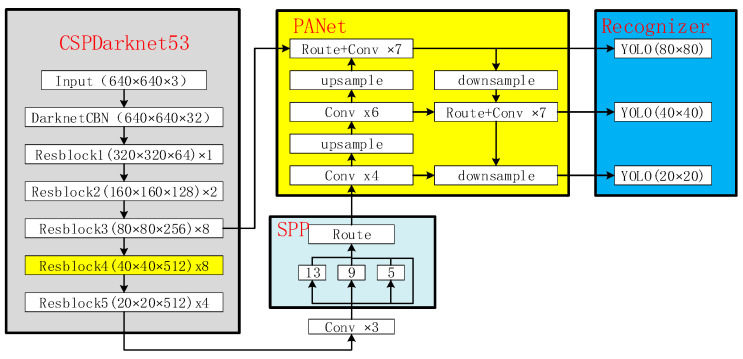
The network structure of YOLOv4.

**Figure 2:**
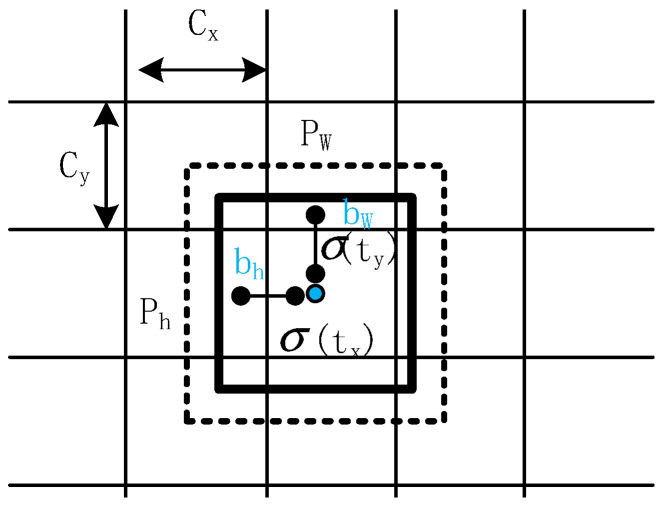
Schematic diagram of location prediction based on anchor.

YOLOv4 was completed by Alexey Bochkovskiy in 2020. It is an end-to-end one-stage network. Its network structure is shown in Figure 1. Firstly, the input image is resized into a multiple of 32, such as 640 × 640. Multi-scale feature maps are extracted of 20 × 20, 40 × 40, and 80 × 80 through the backbone network CSPDarknet53. The obtained feature map is divided into S × S grid cells, and each grid of the feature map is a predicted BBox, as shown in Figure 2. Each BBox prediction bounding box [26] contains location information $\left(t_{x},t_{y},t_{w},t_{h}\right)$ and confidence ($t_{0}$), in which $t_{x},\;\;t_{y}$ are mapped between 0 and 1 through the sigmoid function, and the prediction center is fixed in the cell, making the model more stable. $c_{x}$, $c_{y}$ represent the distance between the cell and the upper left coordinate of the characteristic diagram, $p_{w}$, $p_{h}$ represent the width and height of the anchor, and $\left(b_{x},b_{y},b_{w},b_{h}\right)$ represents the prediction result based on the anchor. The calculation formula is shown in Equation (1), where σ represents the sigmoid function.

(1) $$\{\begin{array}{c} b_{x}=\sigma \left(t_{x}\right)+c_{x}\\ b_{y}=\sigma \left(t_{y}\right)+c_{y}\\ b_{w}=p_{w}e^{t_{w}}\\ b_{h}=p_{h}e^{t_{h}} \end{array}$$

### 2.2. The Improved YOLOv4

YOLOv4 is mainly used for target recognition of conventional size from the head-up perspective. Using YOlOv4 for direct detection wastes computing resources for detecting large targets and is easy to lose the details of small targets. The improvement starts from the network depth and network width, and a backbone network is designed suitable for small target recognition to improve the accuracy of the network.

Although the over-deep network can extract more advanced semantic information, which has great advantages in dealing with large data sets, it will cause the loss of target spatial information, that is, it is difficult to complete the target positioning due to the lack of target details. Therefore, the original backbone network is improved for the problem that the loss of target detail information leads to low accuracy of the algorithm. Firstly, aiming at the problem of small target information loss, the residual block Resblock4, which is mainly used for medium target feature extraction in the original backbone network of Figure 1, is discarded. Secondly, in the Path Aggregation Network (PANnet) [27], the feature maps are downsampled and upsampled three times. Although this process finally restores the feature map to the original size, the restoration process is completed by linear interpolation, which causes the feature map to become blurred. Therefore, the detector is appropriately advanced, to enhance the ability of the network to detect small targets. Finally, to improve the recognition accuracy, an SPP module is added to the network, and 10 convolutional layers are used for feature extraction afterward.

**Figure 3:**
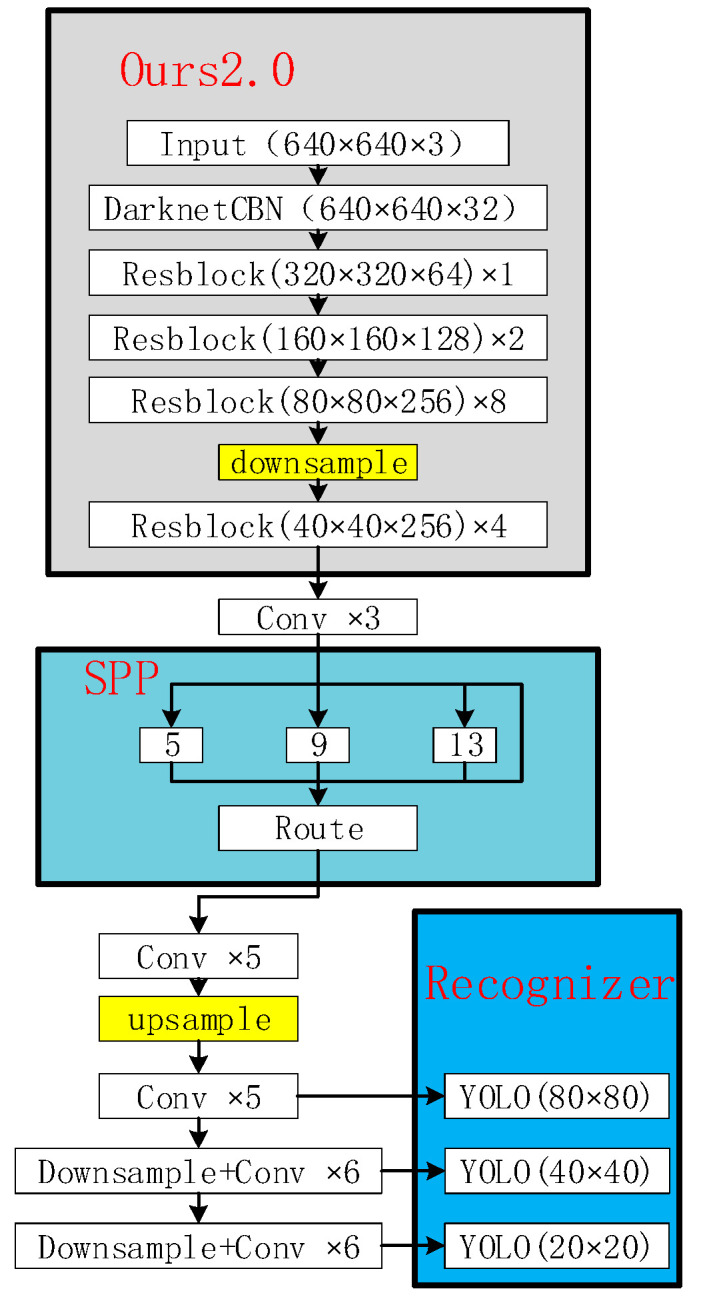
The structure of the improved network.

On the other hand, for the problem that the network width greatly increases the net-work computation amount. Firstly, the number of channels of Resblock5 in Figure 1 is reduced from 512 to 256, which can reduce the calculation amount of the Resblock by a factor of four. Secondly, the SPP module quadruples the number of channels, resulting in a sharp increase in the amount of computation and parameters of a convolutional layer behind the SPP module. Therefore, the size of the feature map is changed from the original 80 × 80 to 40 × 40 through a downsample before the SPP module, so that the calculation amount between downsample and upsample is reduced by a factor of four. The improved YOLOv4 network structure is shown in Figure 3.

The improved algorithm is tested based on the VisDrone dataset [28], and the test results are shown in Table 1. The test results show that the parameters of the improved backbone network are reduced to 10.4% of the original network, the accuracy is improved by 2.7%, and the reasoning speed is 1.8 times that of the original network in the task of small target recognition from the perspective of UAV. However, due to the network improvement, only the number of channels is designed for the entire Resblock, and there is still a redundancy problem in the network.

To solve the problem of a large amount of calculation and slow reasoning speed caused by redundant channels in the network, the redundant channels are pruned based on BN layer coefficients. The BN layer standardizes the input of each layer of the network so that the data distribution shifts to the mean and variance of the overall data, which makes the network easier to initialize and accelerates network training. The BN layer preserves the sample characteristics through two learnable parameters to improve the flexibility of the network. The process of the BN algorithm is shown in Formula (2), where $x_{i}$, $y_{i}$ are the input and output samples, $\mu_{ℬ}$, $\sigma_{ℬ}^{2}$ are the mean and variance of batch data, and γ, β are the learnable scaling factor and shift factor.

(2) $$\{\begin{array}{c} \mu_{ℬ}=\frac{1}{m}\overset{m}{\underset{i=1}{\sum }}x_{i},y_{i}\\ \sigma_{ℬ}^{2}=\frac{1}{m}\overset{m}{\underset{i=1}{\sum }}{\left(x_{i}-\mu_{ℬ}\right)}^{2}\\ {\hat{x}}_{i}=\frac{x_{i}-\mu_{ℬ}}{\sqrt{\sigma_{\beta }^{2}+\varepsilon }}\\ y_{i}=\gamma {\hat{x}}_{i}+\beta ={\mathrm{BN}}_{\gamma ,\beta }\left(x_{i}\right) \end{array}$$

It can be seen from Formula (2) that when γ is small, the channel has only a small input to the subsequent module, that is, the module is less important. Redundant channels can be removed by evaluating the importance of channels by sparse γ coefficients. According to this principle, the designed network is pruned, the parameters of the BN layer of the network model are thinned using L1 regularization, and the γ value of the sparse model is sorted. The channel pruning of the network is carried out by using the strategy of global pruning of 54% of the parameter quantity and at least 10% of the single layer, the network test results after pruning are shown in Table 2. By pruning redundant channels, the amount of network parameters is reduced to 32.1% of the original, and the amount of calculation is reduced to 4.3% of the original. When the network loses only 0.9% of the accuracy rate, the network inference speed reaches 1.9 times that before pruning.

After the pruning is completed, to further improve the inference speed of the algorithm, TensorRT is used to quantify and accelerate the network. The pruning quantification test results are shown in Table 3. After accelerating the improved network with TensorRT, the network accuracy is only reduced by 0.3%, the inference speed is increased by 26%, and the performance of the algorithm on the embedded platform is greatly enhanced.

Overall, compared with the original algorithm, the accuracy of the improved algorithm is increased by 1.5% and the speed is increased by 3.3 times.

## 3. Tracking Algorithm Based on KCF

### 3.1. Basic Principles of KCF

The basic idea of the tracking method based on correlation filtering is to design a filtering template according to the first frame sample, and the template will perform a correlation operation on the image of the search area. The maximum response position is the current target position, as shown in Formula (3), where g represents the response output, f represents the input image, h represents the filter template, and ∗ represents the convolution operation.

(3) g=f∗h

To improve the operation speed, the convolution in the time domain can be transformed into dot multiplication in the frequency domain. As shown in Formula (4), ℱ represents Fourier transformation, and ⊙ represents point multiplication. The core task of correlation filtering is to find the optimal filtering template.

(4) $$\{\begin{array}{c} F=F\left(f\right)\\ H=F\left(h\right)\\ G=F⊙H^{*} \end{array}$$

MOSSE, the first tracking algorithm based on correlation filtering, was proposed by Bolme D S et al. In 2010. Firstly, the filter template is designed through a set of training images $F_{i}$ and expected output $G_{i}$. As shown in Formula (5).

(5) $$H_{i}^{*}=\frac{G_{i}}{F_{i}}$$

For the whole image sequence, the algorithm hopes to minimize the square sum of the error between the true value and the predicted value of all sequences and solve the $H^{*}$ to obtain the template. Such as Formula (6), $\underset{i}{\sum }F_{i}⊙F_{i}^{*}$ represents the energy spectrum of the input image.

(6) $$\{\begin{array}{c} \underset{H^{*}}{min}\underset{i}{\sum }{\left|F_{i}⊙H^{*}-G_{i}\right|}^{2}\\ H^{*}=\frac{\sum_{i}G_{i}⊙F_{i}^{*}}{\sum_{i}F_{i}⊙F_{i}^{*}} \end{array}$$

Due to the illumination change, rotation, and other transformations in the tracking process, the filter template needs to be updated online to adapt to the changes in the environment. The update strategy is shown in Formula (7), in which η is the learning rate, which makes the weight of the current frame larger, and the influence of the past frame attenuates with time.

(7) $$\{\begin{array}{c} H_{i}^{*}=A_{i}/B_{i}\\ A_{i}=\eta G_{i}⊙F_{i}^{*}+\left(1-\eta \right)A_{i-1}\\ B_{i}=\eta F_{i}⊙F_{i}^{*}+\left(1-\eta \right)B_{i-1} \end{array}$$

KCF kernel correlation filtering algorithm has a very high speed and accuracy. Compared with MOSSE, KCF uses the HOG feature to replace the original pixels and proposes a multi-channel training strategy, which performs better in the case of illumination change and enhances the robustness of the algorithm. In the training stage, the nonlinear problem is introduced into the high-dimensional space through the kernel function to make it linearly separable. The filter is trained by ridge regression. The objective function is as shown in Equation (8), w is the filter template, x is the input image, y is the true value, and ∗ represents the correlation operation.

(8) $$\underset{w}{\min}{\left(x*w-y\right)}^{2}+\lambda ∥w∥{}^{2}$$

Introduce Formula (8) into Fourier transform, as shown in Formula (9). The solution of $\hat{w}$ can be obtained by deriving $\hat{w}$ and making its value 0. The result is shown in Formula (10). KCF uses a kernel function to map input features to high-dimensional space, such as Formula (11). The Formula (11) is introduced into the frequency domain and the derivative of α is 0 to obtain the optimal solution, as shown in Formula (12), where ${\hat{k}}^{xz}$ is the kernel correlation matrix.

(9) $$\underset{\hat{w}}{\min}{\left(\hat{x}⊙{\hat{w}}^{*}-\hat{y}\right)}^{2}+\lambda ∥\hat{w}∥{}^{2}$$

(10) $$\hat{w}=\frac{\hat{x}⊙\hat{y}}{\hat{x}⊙{\hat{x}}^{*}+\lambda }$$

(11) $$\{\begin{array}{c} w=\underset{w}{min}{\left(\emptyset {\left(X\right)}^{*}w-y\right)}^{2}+\lambda ∥w∥{}^{2}\\ w=\underset{i}{\sum }\alpha_{i}\emptyset \left(x_{i}\right)\\ \alpha =\underset{\alpha }{min}\emptyset \left(X\right)\emptyset {\left(X\right)}^{T}\alpha -y^{2}+\lambda ∥w∥{}^{2} \end{array}$$

(12) $$\hat{a}=\frac{\hat{y}}{{\hat{k}}^{xz}+\lambda }$$

In the process of tracking the target, the appearance of the target will change due to scale, attitude, and light and shadow, so the classifier needs to be updated in real-time. As shown in Formulas (13) and (14), where ${\hat{\bar{a}}}_{t}$ is the update coefficient of the classifier, ${\hat{\bar{x}}}_{t}$ is the appearance of the target, and η is the update rate.

(13) $${\hat{\bar{a}}}_{t}=\eta {\hat{a}}_{t}+\left(1-\eta \right){\hat{a}}_{t-1}$$

(14) $${\hat{\bar{x}}}_{t}=\eta {\hat{\hat{x}}}_{t}+\left(1-\eta \right){\hat{\bar{x}}}_{t-1}$$

### 3.2. Improvement of KCF

#### 3.2.1. Adaptive Learning Rate

KCF algorithm has the advantages of fast speed, low requirements for hardware platform, online template updating, and can adapt to the changes of objectives to a certain extent. However, in the application scenario of UAV tracking, there are some problems such as target occlusion and scale transformation. Aiming at the poor robustness of KCF in UAV tracking, an improved KCF algorithm with an adaptive learning rate is proposed.

Because KCF adopts a fixed learning rate, the tracking template will be polluted when the tracking target is blocked, similar targets interfere, and the tracking background is complex. This paper judges the tracking situation based on the tracking response parameters reduces the learning rate when the tracking is poor, avoids the template from being polluted, and realizes a highly robust update through an adaptive learning rate. Reference [29] mentioned a method to judge occlusion based on the response matrix. As shown in Formula (15), $F_{max}$, $F_{min}$ is the maximum and minimum value in the response matrix, and $F_{w,h}$ is the response value at (w, h) in the response matrix.

(15) $$APCE=\frac{F_{max}-F_{min}}{mean\left(\sum_{w,h}{\left(F_{w,h}-F_{min}\right)}^{2}\right)}$$

**Figure 4:**
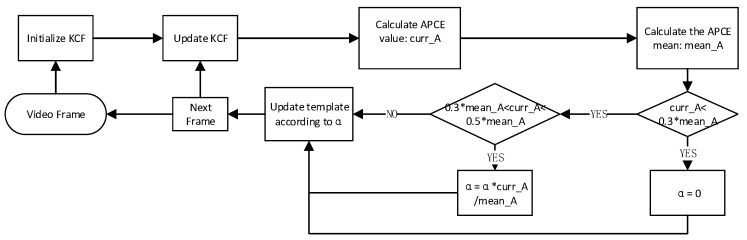
The flow of APCE based high confidence update algorithm.

However, due to different types of tracking targets, large differences in background complexity, different moving speeds of targets, and other factors, the APCE value will be different, so it is difficult to use a single threshold to judge the tracking situation of different sequences. Therefore, given the defects of APCE, based on the idea of data filtering, this paper proposes a method of adaptive APCE threshold for each sequence. The specific process is shown in Figure 4. First, the APCE mean of each sequence is calculated, and the outliers are eliminated. Then, whether to update the template is determined by the relevant threshold.

**Figure 5:**
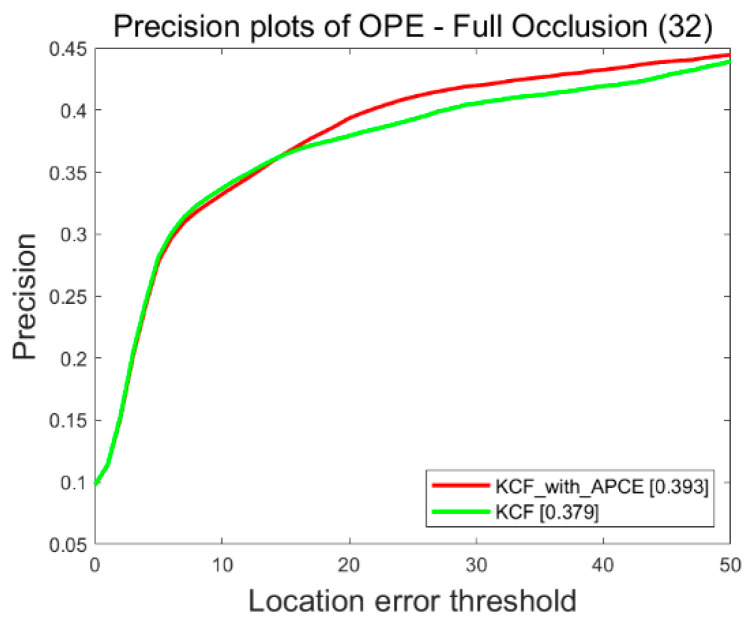
Experimental results of adaptive learning rate.

Algorithms are compared on objects’ occluded sequences of dataset UAV123. The one-pass evaluation method (OPE) [30] is used for experiments. This method initializes the first frame with the position of the target in the true value, then runs the tracking algorithm to obtain the algorithm result and calculates the average precision and success rate through the set threshold. In this section, the accuracy map is used to evaluate the results, and the experimental results are shown in Figure 5. The experimental results show that the speed of the improved algorithm is consistent with the original algorithm, and the accuracy of the improved algorithm is 1.4% higher than the original algorithm.

#### 3.2.2. Scale Improvement

KCF has certain advantages in accuracy and speed, but the algorithm does not update the scale. When the size of the tracking target changes, the algorithm cannot adapt to the size of the target, resulting in tracking failure. In response to the appealing problem, and to ensure the real-time performance of the algorithm, this paper adopts three scale pools (1.05, 1, 0.95) to adapt to the change in target scale. In each frame of tracking, three scales are used for tracking. To adapt to the rapid changes of the target, the three basic scales are transformed using an adaptive scale transformation factor to achieve the effect of multiple scale pooling.

Record the target scale changes of five consecutive frames, when the frame of the target scale becomes larger, it is marked as 1, when the frame of the target scale becomes smaller, it is marked as −1, and the unchanged frame is marked as 0. Add the five numbers, convert the absolute value of their sum, and multiply it with the default scale transformation factor to obtain the scale adjustment factor of the current frame. When the target continuously becomes larger and smaller, the change rate can be automatically adjusted to achieve fast and accurate scale change. The calculation formula is shown in Formula (16), in which scales are the current scale pool of the scale, $s_{0}\cdots s_{4}$ is the scale transformation of the previous 5 frames of the record number. When the suppressed response peak is still larger than the response peak of the original scale, the scale is updated to obtain the scale with the largest response value as the new scale. After the tracking scale has not changed for eight consecutive frames, the scale is adjusted to the initial scale.

(16) $$scales=scales\times \left(1+\frac{\sqrt{{\left(s_{0}+\cdots +s_{4}\right)}^{2}}}{10}\right)$$

**Figure 6:**
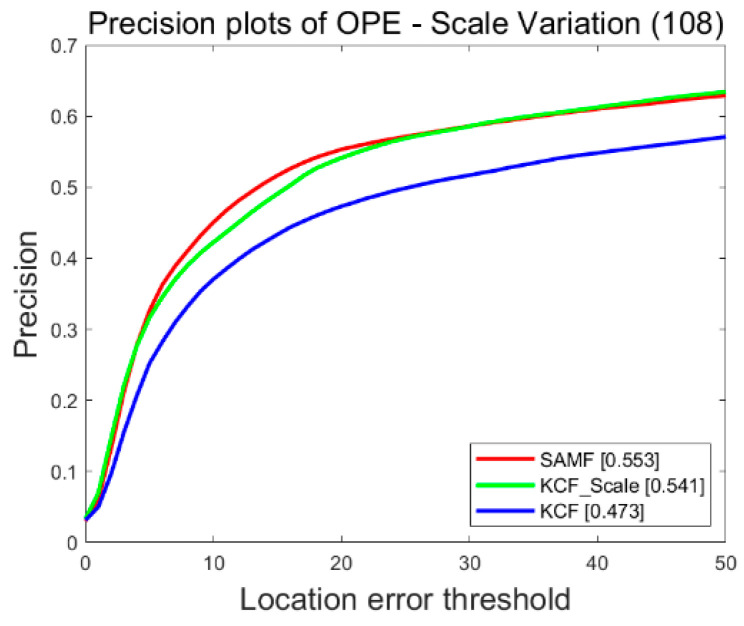
Experimental results of scale improvement.

The improved algorithm is tested by the UAV123 dataset, and the experimental results are shown in Figure 6. The experimental results show that, through the adaptive scale, three basic scales can be used as six changed scales, and the improved algorithm improves the accuracy by 6.8% compared with the original KCF.

#### 3.2.3. Target Re-Recognition

**Figure 7:**
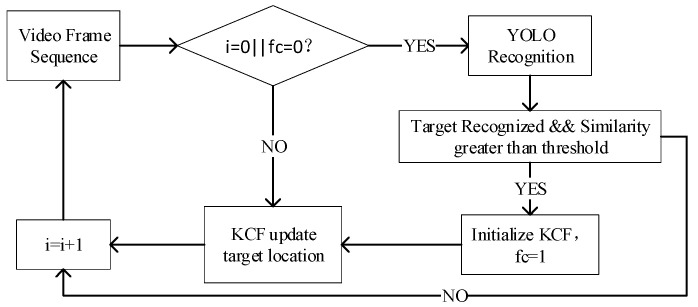
Autonomous initialization tracking combined with object recognition.

To improve the robustness and intelligence of the tracking algorithm, the autonomous initialization and intelligent re-recognition of the target are realized by combining the target recognition algorithm and the image similarity recognition algorithm. The flow chart is shown in Figure 7.

In Figure 7, *i* is the video frame sequence number and *fc* is the flag bit of whether the KCF tracking is successful. When the tracking fails according to the response, *fc* is set to 0. At the beginning of the algorithm, *i* = 0, the target is recognized through the YOLOv4 target recognition algorithm. Calculate the similarity between the results of the same category as the target of interest and the pre-stored target image through the color histogram, use the recognition result with the largest similarity to initialize the KCF, and set the tracking flag to 1 to start tracking continuously. When the tracking fails, the initialization operation is carried out again.

**Figure 8:**
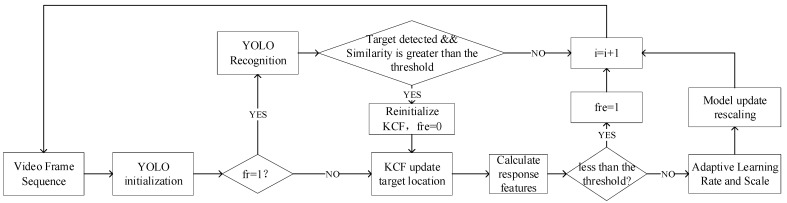
Intelligent re-tracking algorithm with target re-recognition.

In the tracking process, there are situations such as the target is blocked, the target moves rapidly, etc., which causes the tracking to fail. To achieve stable tracking of the target, it is necessary to combine the information before the target disappears to re- recognition and continue to track. By integrating the target recognition algorithm, the algorithm flow is shown in Figure 8. After the target is completely occluded, take the place where the target disappears as the center, and target recognition searches in an area four times the size of the target. When the target is detected and the similarity is greater than the threshold, the target recognition result is used to re-initialize the tracking target, and again to track.

To verify the effectiveness of the algorithm, experiments were carried out on sequences such as BlurBody, Human6, Jogging1, Jogging2, etc. in the OTB100 dataset. The experimental results are shown in Table 4.

The experimental results show that by integrating the target recognition algorithm, the algorithm can re-recognize the tracking target when it is occluded or moves rapidly, and the accuracy rate is increased by 52.6% on average in the four data sequences, which has good usability.

## 4. Experiment

### 4.1. Tracking System

**Figure 9:**
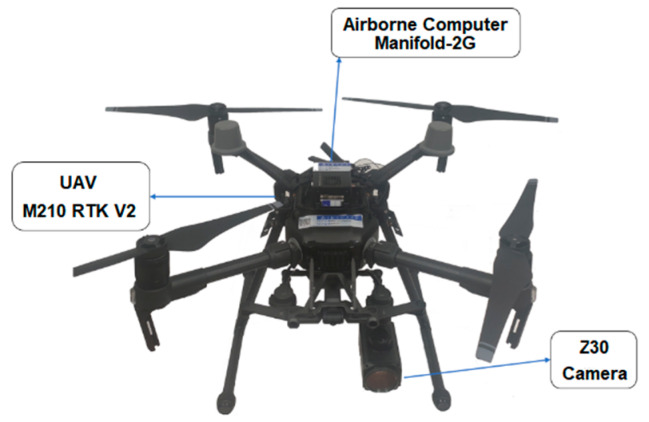
Experimental UAV platform.

To verify the effectiveness of the algorithm, the Onboard SDK (OSDK) is used to control the UAV based on DJI M210, DJI Z30 camera, airborne computer Manifold-2G, and other hardware. The completed hardware platform is shown in Figure 9.

**Figure 10:**
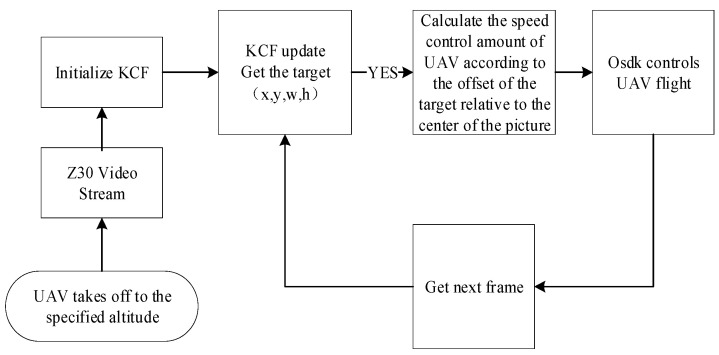
Experimental procedures.

To verify the effectiveness of the algorithm, the experimental flow as shown in Figure 10 is designed. The specific process is as follows:

(1) The UAV will take off autonomously from the take-off point to an altitude of 10 m and 3 m away from the take-off point, and run the improved YOLOv4 algorithm;
(2) Based on the target recognition results, in the process of tracking the target, the similarity between all targets detected by YOLOv4 and the reserved target template is calculated by using the color histogram. To ensure the stability of the algorithm, the target with the highest similarity and the distance from the center of the picture is less than a certain threshold is regarded as the interest target in the experiment;
(3) The coordinate information of the target is used to initialize the improved KCF. After the KCF tracks the target, the pixel value of the target center deviating from the image center is used as the control amount, which is calculated by PID as the speed control amount, and the target is actually tracked.
(4) In the process of tracking, the APCE value is used to judge the tracking situation. When the tracking target is lost, the target re-recognition process will intervene.

The target is always in the center of the picture. When the target is not in the center of the picture, the pixel offset is converted into the UAV speed control through PID control, to complete the actual tracking task.

### 4.2. Experimental Result

Firstly, the intelligent tracking algorithm is tested, and a building corridor is selected as the experimental site. The scene contains pillars. When the tracked target passes through the area, it will be blocked by the pillar, and the tracked target will completely disappear from the picture.

**Figure 11:**
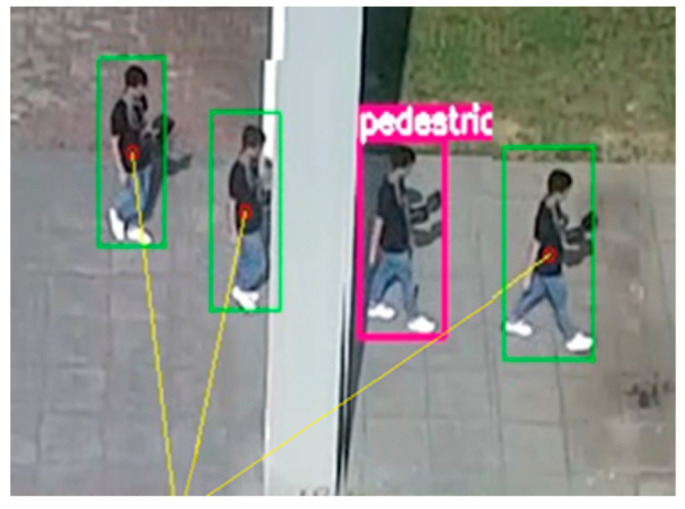
The experiment of automatic initialization and intelligent re-recognition.

The experimental effect is shown in Figure 11. When the target walks behind the pillar and the target is blocked, the algorithm judges the tracking failure through the response value and enters the re-initialization process. Then it starts target recognition, recognizes the target, and combines the position before the target disappears. When the similarity is greater than the threshold, it re-initializes the tracker and continues to track the target. The experimental results show that the designed algorithm can achieve accurate target recognition, automatic initialization, and robust tracking.

**Figure 12:**
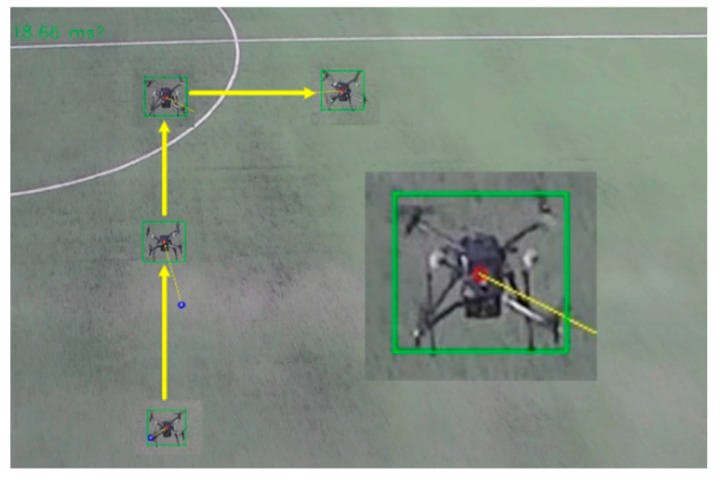
The experiment of tracking UAV.

**Figure 13:**
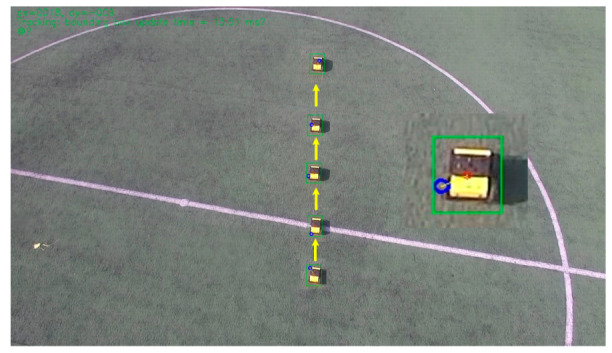
The experiment of tracking UGV.

Secondly, experiments were carried out on the tracking of various types of small targets, and the tracking of UAVs and smart cars were carried out respectively. Figure 12 shows the platform tracks a DJI M210 multi-rotor UAV. In the 1920 × 1080 pixel image, the target only occupies 69 × 63 pixels. The target trajectory is shown by the yellow arrow. In Figure 13, the UAV has tracked a ground unmanned vehicle, which occupies only 39 × 54 pixels in the picture. The tracking results show that the platform and methods can stabilize and effectively track small targets.

**Figure 14:**
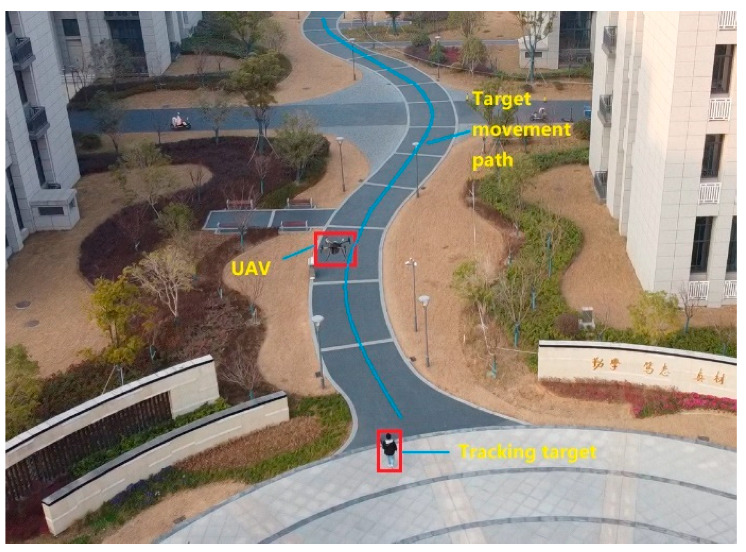
The scene of the tracking experiment.

**Figure 15:**
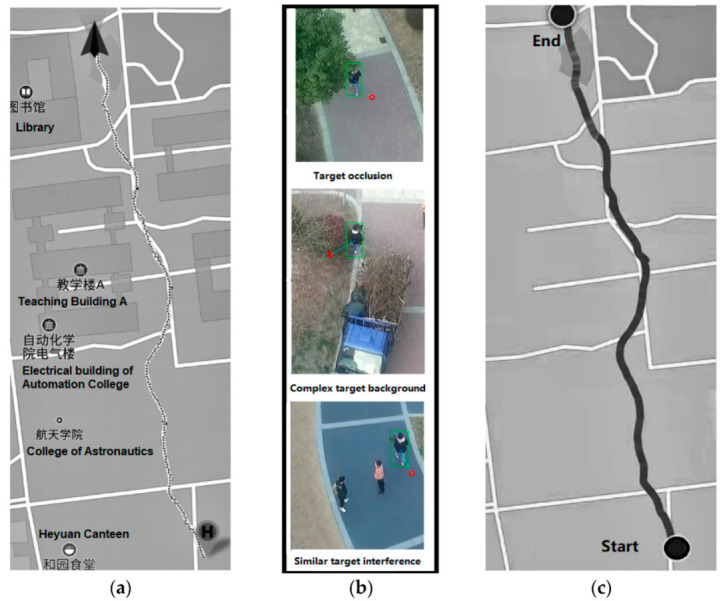
Target tracking in complex environment (**a**) UAV trajectory; (**b**) Real-time tracking image of UAV; (**c**) Target trajectory.

Finally, the target tracking in a complex environment is tested, and a pedestrian in the path between tall buildings is tracked. As shown in Figure 14, during the whole tracking process, the UAV flew at a height of 16 m and a tracking distance of 493 m, which took 7 min and 13 s, and the average speed was 1.14 m/s. The tracking trajectory is shown in Figure 15.

In the tracking of a complex environment, the tracked target is a pedestrian, and the trajectory of the UAV is consistent with that of the tracked target of interest. When multiple pedestrians appear in the picture at the same time, the algorithm can eliminate the interference of similar targets and track the specific pedestrian. In the complex background, the algorithm updates the template adaptively to avoid the pollution of the tracking template and realizes the target tracking in the complex environment. In addition, when the target is blocked, it can still be tracked again by the re-recognition algorithm, which shows the characteristics of strong robustness and high level of intelligence of the algorithm.

## 5. Conclusions

An improved algorithm for a lightweight network for target recognition is proposed. Backbone network, redundant channels are pruned based on the BN layer coefficients, and TensorRT is used to accelerate the network, which improves the accuracy and speed of small target recognition. At the same time, aiming at the shortcomings of low robustness and intelligence of the existing KCF tracking algorithm, an improved KCF algorithm with an adaptive learning rate is proposed. The APCE is calculated based on the target response value, and a threshold is set to judge the tracking situation. The template adaptive learning rate update is realized, and the tracking template pollution problem caused by the fixed learning rate is solved. Besides, adaptive scale updating is used to greatly improve the accuracy of the algorithm. Finally, the improved tracking algorithm is combined with the improved target recognition algorithm, which improves the robustness of the algorithm while realizing automatic re-recognition when tracking fails. A physical experimental platform is built up based on DJI M210 UAV. The physical experiment results show that the improved algorithm proposed in this paper realizes real-time, accurate, and highly robust recognition and tracking of small targets.

## Figures and Tables

**Table 1:** Network improvement test results.

Backbone Network	Computation/BFLOPs	Parameters/MB	Map	FPS
CSPDarknet53	141	256.3	34.1%	2.1
Ours2.0	65	26.8	36.8%	3.8

**Table 2:** Network pruning test results.

Backbone Network	Computation/BFLOPs	Parameters/MB	Map	FPS
Before pruning	65	26.8	36.8%	3.8
After pruning	28	8.6	35.9%	7.3

**Table 3:** Network acceleration test results.

Measurement	Map	FPS
Before acceleration	35.9%	7.3
After acceleration	35.6%	9.2

**Table 4:** Experimental comparison of re-recognition fusion algorithms.

Data Sequence	BlurBody	Human6	Jogging1	Jogging2
KCF accuracy	58.4%	29.0%	23.5%	54.3%
Fusion algorithm accuracy	95.5%	94.0%	94.7%	91.4%

## Data Availability

We used publicly available datasets in this study. VisDrone dataset at https://doi.org/10.1109/ICCVW.2019.00031 (accessed on 7 August 2022).

## Abbreviation

UAV	Unmanned Aerial Vehicle
KCF	Kernel Correlation Filter
YOLO	You Only Look Once
SIFT	Scale Invariant Feature Transformation
HOG	Histogram of Oriented Gradients
CNN	Convolutional Neural Network
R-CNN	Regions with Convolutional Neural Network Features
SSD	Single Shot multi-box Detector
DCFNet	Discriminant Correlation Filters network
ECO	Efficient Convolution Operators for tracking
SiamFC	Fully-Convolutional Siamese networks
MOOSE	Minimum Output Sum of Squared Error
CSK	Circulant Structure with Kernels
BBox	Bounding Box
BN	Batch Normalization
SPP	Spatial Pyramid Pooling
APCE	Average Peak-to-Correlation Energy
OPE	One-pass Evaluation
